# Antimicrobial stewardship programs in a Mexican private healthcare system: a self-assessment of core elements

**DOI:** 10.1186/s12879-024-09601-9

**Published:** 2024-11-06

**Authors:** José Iván Castillo Bejarano, Dzoara Laura Lugo Ondarza, Juan O Galindo Galindo, Daniel Siller Rodríguez, Sara Paulina Rosales-González, Susana Patricia Cantú González, Jorge Alberto Vera Delgado

**Affiliations:** 1 Hospital Epidemiological and Surveillance Unit, Christus Muguerza Alta Especialidad, Monterrey, 64060 ZC México; 2 Epidemiology and Infection Control Manager. Christus Muguerza Healthcare System, Monterrey, 64060 ZC México; 3 Christus Muguerza Healthcare System, Monterrey, 64060 ZC México; 4 Infectious Diseases Service, University Hospital “Dr José Eleuterio González, Universidad Autónoma de Nuevo León, Monterrey, 66640 México; 5 Christus Muguerza Alta Especialidad, Monterrey, 64060 ZC México

**Keywords:** Antimicrobial resistance, Antimicrobial stewardship, Antimicrobial stewardship programs, Mexico

## Abstract

**Background:**

Antimicrobial stewardship programs (ASPs) refer to a set of coordinated actions that improve the quality of care and combat antimicrobial resistance. Currently, information regarding the status of ASPs in Mexico is scarce. We aimed to describe the status of ASPs in 12 hospitals from Christus Muguerza Healthcare System.

**Methods:**

A cross-sectional study was conducted in 12 hospitals, with a previously developed self-assessment tool to calculate each hospital’s ASP development score. The self-assessment tool includes 7 standards with 23 items. Score categories were defined as; high, medium, low, or none. The overall ASP development score was calculated using the proportional weight of each standard. Participating hospitals were divided into 2 groups according to their bed count. Statistical analysis was conducted in Excel program (Microsoft, Redmont, Washington).

**Results:**

12 hospitals completed the self-assessment survey. The median overall ASP development score was 32.3%. The highest overall development scores were observed for hospitals with > 40 beds. The core elements with the lowest development scores were Education and training, and Reporting and feedback. Unlike hospitals with over 40 beds, those with 40 beds or less had a low development score for Hospital leadership support. The core element with the highest development score was Infection prevention and control.

**Conclusions:**

This is the first multicenter assessment of ASPs in Mexico, revealing a high proportion of low-score hospitals. National implementation of ASPs is required to combat antimicrobial resistance.

## Introduction

According to data reported in “The Review on Antimicrobial Resistance” chaired by Jim O´Neill, it is projected that antimicrobial resistance (AMR) could lead to 10 million deaths in 2050 [1]. In addition, AMR represents a negative impact on financial implications to healthcare systems and disability-adjusted life-years [2]. A study made in México showed that in the last decade, there has been an increase in resistance for some antibiotics in different bacterial species in Mexico which highlights the need to monitor and combat this trend [3]. To combat AMR the Antimicrobial Stewardship Programs (ASPs) are one of the most important actions in hospital settings, this ASPs represent coordinated actions to reduce antibiotic consumption.

The Center for Disease Control and Prevention and WHO have identified some key elements for ASPs. However, according to a recent survey, most Latin American countries do not have an ASP implemented or a plan to combat AMR [4]. A recent study of twenty hospitals in Latin America reported that there are several barriers for the implementation of ASPs such as lack of hospital leadership support, inadequate staffing and tools, and limited training opportunities [5]. To standardize the status of ASPs a quantitative standardized tool was adopted from ICONTEC (Colombian Institute of Technical Standards and Certification), and JIC (Joint Commission International) accreditation standards to evaluate seven hospitals from Latin American countries, where it was described an average score of 65.8% (range 40-94.3%), with a significant low score to items related to monitoring a communicating the ASPs progress [6].

Our national situational diagnosis of ASPs programs is scarce and has been limited to the participation of single centers in multicentric studies [6]. In similar studies worldwide, Mexico tends to be under-represented which has led to the limited information we have up to date. Christus Muguerza Healthcare System is a group of private hospitals distributed in Mexico, where to combat the AMR problem an ASPs standard was implemented in all hospital settings. In our work, we aimed to describe the development status of ASPs in 12 hospitals in Mexico.

## Materials and methods

This is an observational, transversal, cross-sectional and multicenter study conducted a in 12 hospitals from Mexico. These hospitals comprise the Christus Muguerza Healthcare System in Mexico located in seven states. These are academic-private institutions from urban areas. Table 1 displays the basic characteristics of hospitals. To depict the ASPs status the participating hospitals were divided into 2 groups according to their bed count: (1) Group 1, 1–40 beds; (2) Group 2, > 40 beds, according to the mean number of beds for a hospital reported in Mexico.

**Table 1:** Hospital characteristics and score of the antimicrobial stewardship program self-assessment

Hospital	Location	Beds	Hospital leadership support (3 items/ weight 5%)		AMS comittee / team structure (5 items/ weight 15%)		Infection prevention and control (5 items/ weight 20%)	Education and training (2 items/ weight 10%)		Action (3 items/ weight 20%)		Tracking (3 items/ weight 20%)		Reporting and feedback (2 items/ weight 10%)		Overall ASP assessment		Median		Q1		Q3
Christus Muguerza Hopital Alta Especialidad	Nuevo León	113	44,0%		59,8%		100,0%	66,5%		33,0%		55,3%		66,0%		62,1%		59.8%		44%		66.5%
Christus Muguerza Hospital Saltillo	Coahuila	80	33,0%		0,0%		72,8%	0,0%		0,0%		11,0%		0,0%		18,4%		0.0%		0.0%		33%
Christus Muguerza Hospital Sur	Nuevo León	73	0,0%		0,0%		93,2%	0,0%		66,3%		77,3%		0,0%		47,4%		0.0%		0.0%		77.3%
Christus Muguerza Hospital Conchita	Nuevo León	67	75,3%		59,8%		100,0%	16,5%		11,0%		21,0%		48,0%		45,6%		48.0%		16.5%		75.3%
Christus Muguerza Hospital del Parque	Chihuahua	64	55,0%		73,2%		86,4%	33,0%		44,0%		44,3%		0,0%		52,0%		44.3%		33.%		73.2%
Christus Muguerza Hospital del Faro	Yucatán	48	11,0%		19,8%		100,0%	0,0%		11,0%		44,3%		0,0%		34,6%		11.0%		0.0%		44.3%
Christus Muguerza Hospital UPAEP	Puebla	36	11,0%		39,8%		73,2%	0,0%		0,0%		44,3%		0,0%		30,0%		11.0%		0.0%		44.3%
Christus Muguerza Hospital Reynosa	Tamaulipas	31	33,0%		19,8%		93,2%	0,0%		11,0%		11,0%		0,0%		27,7%		11.0%		0.0%		33%
Christus Muguerza Hospital Altagracia	Guanajuato	30	0,0%		0,0%		73,0%	16,5%		22,0%		0,0%		0,0%		20,7%		0.0%		0.0%		22%
Christus Muguerza Hospital Vidriera	Nuevo León	29	33,0%		66,4%		100,0%	0,0%		33,0%		11,0%		0,0%		40,4%		33.0%		0.0%		66.4%
Christus Muguerza Hospital San Nicolas	Nuevo León	20	0,0%		0,0%		59,8%	0,0%		11,0%		11,0%		0,0%		16,4%		0.0%		0.0%		11%
Christus Muguerza Hospital San Pedro	Nuevo León	8	0,0%		6,6%		79,8%	0,0%		22,0%		22,0%		0,0%		25,8%		6.6%		0.0%		22%
Hospital characteristics and score of the antimicrobial stewardship program self-assessment according to the number of beds.																						
				Hospital leadership support		AMS committee / team structure			Infection prevention and control		Education and training		Action		Tracking		Reporting and feedback		Overall ASP assessment			
All hospitals				22,0%		19,8%			89,8%		11,0%		16,5%		21,5%		9,5%		32,3%			
All hospitals median				22,0%		19,8%			89,8%		0,0%		16,5%		21,5%		0,0%		32,3%			
All hospitals Q1				0,0%		0,0%			73,2%		0,0%		11,0%		11,0%		0,0%		24,5%			
All hospitals Q3				35,8%		59,8%			100,0%		16,5%		33,0%		44,3%		0,0%		46,0%			
1–40 beds				5,5%		13,2%			76,5%		0,0%		16,5%		11,0%		0,0%		26,7%			
> 40 beds				38,5%		39,8%			96,6%		8,3%		22,0%		44,3%		0,0%		46,5%			

The self-assessment was conducted using a standardized tool published by Christian Pallares et al. [6]. This tool includes seven standards with 23 items. The core elements evaluated are: (1) Hospital leadership support, (2) Antimicrobial stewardship (AMS) committee / team structure, (3) Infection prevention and control, (4) Education and training, (5) Action, (6) Tracking, and (7) Reporting and feedback. In an initial questionnaire each facility answers in a binary mode (yes or no) based on Table 1 from Pallares´research [6]. The results for each standard were reported in four categories; high = 100%, medium = 66%, low = 33%, or none = 0% depending on the current status of the item. Regarding ASPs status, hospitals were categorized according to the overall ASPs assessment as; (1) basic level (< 33%), (2) intermediate level (≥ 33% - ≤66%), and (3) advanced level (> 66%). To collect the information, a group of ASPs experts meeting from each hospital was conducted face-to-face. The group of experts is represented by physicians working in the infection prevention and epidemiology department. The same group of experts evaluated the information shown by the leaders of each one of the hospitals included in this study. The group of experts who made the self-assessment works in Christus Muguerza healthcare system. Statistical analysis was conducted in Excel program (Microsoft, Redmont, Washington).

## Results

According to the diagnostic tool implemented in 12/12 hospitals with a median of 42 beds, the median of ASP-development score was 32.3% (IQR, 24.5-46.0%). The highest ASP development was observed in group 2 with a median of 46.5%, see Table 1), while group 1 displayed the lowest median score 426.7%. Regarding ASPs status, none of the groups reached the advanced level, group 2 was categorized as intermediate level (46.5%), while group 1 basic level (26.7%). Of note, no hospital reached the advanced level, being a median score of 59.8% the highest of our results.

**Fig. 1:**
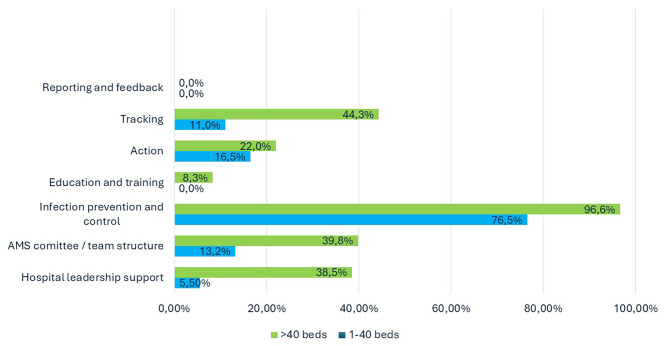
Antimicrobial stewardship program development scores for each core element, stratified by groups

The standards with the lowest score were reporting and feedback with 9.5%, followed by education and training (11%, see Fig. 1). On the other hand, the highest development scores were those related to infection prevention and control (86%) and tracking (29%). For hospital leadership support reflecting ASPs as part of the institutional goal, assignation of institutional budget, and knowing the national standard by hospital leaders the study exhibited results as low as 0% in four hospitals, with a range as high as 75.3% in hospitals with > 40 beds. According to the standard of ASPs committee and team structure, we revealed a wide variation from 0 to 73.2%, where group 1 showed a median of 13.2% in comparison to 39.8% in group 2.

Regarding the standard of actions referring to activities such as “antimicrobial time out” showed a low performance with an median of 16.5%. As previous standards, group 2 exhibited a highest score with 22.0%, followed by group 1 (16.5%).

## Discussion

The present study showed the status of ASPs in 12 hospitals in Mexico, revealing a wide variation of scores between items and basic overall development (32.3%). Data regarding ASPs programs in Mexico is scarce and has only been described in single centers as a part of multicentric studies [6]. Our study highlights the need for more resources to establish ASPs in our country to combat the AMR problem.

Median overall score of 32.3% is inferior to previous studies in Latin America (LATAM), where Pallares et al. [6] showed a mean of 65.8% in seven countries (Argentina, Brazil, Chile, Mexico, Costa Rica, Colombia, and Peru); moreover, an increase to 82.3% was described after an intervention to promote the development of the ASPs. In comparison to our hospitals, Pallares’ hospitals were institutions with more than 110 beds, and half of our hospitals belong to group 1 which means less than 40 beds. Of note, another study from Fabre et al. [5] in LATAM revealed a basic level in 35% of hospitals, including countries such as: Panama, Guatemala, Ecuador, Colombia, and Argentina. One hypothesis of these variations could be linked to the differences between private and public institutions’ budgets; hence, Christus Muguerza Healthcare System is a private hospital system we described items with scores as low as 0%.

Reporting and feedback items, followed by education and training had the lowest scores in our study with 9.5% and 11%, respectively. Although these problems were also noted in other studies from LATAM, inadequate training has been reported in other WHO regions [7, 8]. One study made in LATAM showed that the improvement in reporting and feedback was low after the implementation of ASPs. It showed that Argentina, Chile, and Peru had 0% improvement in this standard. The same study showed that in the standard of education and training, only two countries had improvement, which were Argentina with 50% and Peru with 67%. The rest of the countries, Brazil, Chile, Colombia, Costa Rica and Mexico, showed a 0% improvement in education and training [6]. One study from Lebanon exhibited that only 41% of healthcare personal received training on the rationale use of antibiotics, similar to the results reported in Pakistan where 15 out of 17 doctors responded and stated that no educational program existed to educate them about ASPs [8, 9]. These results are replicated in Uganda where 92.9% of hospitals with ASPs reported lack of training and education as a challenge [10].

Other barriers to ASPs such as shortage of human resources and absence of leadership are reported in 11 out of 22 studies according to a recent systematic review, followed by lack of dedicated ASP found in 6 studies [11]. In our study, we observed a wide difference in hospital leadership support from 38.5% in hospitals with > 40 beds compared to 5.5% in < 40 beds. This score has been documented in a similar range from LATAM studies between 50 and 57% [5, 6]. One explanation of LATAM results could be the lack of financial structure to support the human resources needed to promote the AMS committee. However, one study in Nigeria showed a significant reduction in antimicrobial prescriptions despite the lack of funds, leading from an initial 82.5% in 2015 prescription to 51.1% in 2018 [12]. Nigeria achieved the reduction of antimicrobial prescriptions by implementing a strategy that was based on documenting the reason for antimicrobial prescriptions and a stop-review date. This led to a targeted therapy method, which led to this reduction in antimicrobial prescriptions because previously the use of antimicrobials was mostly empirical. [12]

The previous studies have similar barriers as the one we identified in our study, and they showed that an improvement was made by making some strategies. In Nigeria, the documentation in justifying the use of an antimicrobial and the stop review date helped to reduce the prescription of antimicrobials. [12] In Pakistan, they saw in a multicounty study that by following antimicrobial guidelines the use of empirical antimicrobials was reduced [8]. In Lebanon, they had a strategy to have an ID physician or pharmacist rounding. This strategy of asking for approval for restricted antibiotics made the team think more carefully about the treatment options [9].

We described a mean of AMS structure of 19.8% with wide variation from 0 to 73.2%. Fabre et al. [5] reported a 65% score in AMS structure in five LATAM countries; however, only 20% were formal committees and only 10% met more than 4 times a year. The lack of formal committees and reunions is only a representation of the lack of organization and leadership support, which is one of the standards that are studied in most of the ASPs. This lack of support may be the reason for the differences observed in the overall scores compared to other hospitals evaluated in Pallares et al. study which are institutions with more than 110 beds and most of them academic hospitals with a more formal committee. The results from Fabre et al. are lower in comparison to hospitals evaluated in Pallares et al. [6] study with a mean of 76.9%.

The establishment of a dedicated multidisciplinary ASP committee has been reported as a facilitator in establishing ASPs studies from low and middle-income countries [13]. The establishment of AMS committees would lead to the creation of institutional guidelines, a measure that has shown to be cost-effective according to a systematic review that showed a cost reduction of $448 per 100 patient-day [14].

We encounter limitations such as methodological constraints, limited human resources, staff and tools. We shall consider that as a private hospital, we have more resources than a public hospital, however, there is a lack of institutional departments which makes it harder for the ASPs program to exist. We also may face a bias that is based on the knowledge that the current development of IPC (Infection Prevention and Control Programs) in the region is high which would lead to a bias in the level of ASPs, hence this bias will apply to all centers. Although these two programs should be articulated with each other, the instruments developed for their measurement are independent.

## Conclusion

Our work is the first multicentric study in Mexico where the level of ASPs is described. We observed wide variations between items ranging from 0 to 75.3% and a notable higher support for ASP establishment in hospitals with > 40 beds. Education and training programs targeting healthcare providers are essential for fostering a culture of antimicrobial stewardship and promoting adherence to evidence-based guidelines, which is one of the lowest score items.

Given these findings, there is an urgent need to prioritize antimicrobial stewardship initiatives in Mexico. We believe that the use of strategies such as following guidelines, having an ID physician that authorizes the use of antimicrobials, justifying the use of the antimicrobials, and having a stop-review date may help us to improve the use of antimicrobials by supporting the ASPs. Antimicrobial stewardship programs play a pivotal role in optimizing antimicrobial use, promoting judicious prescribing practices, and reducing the selective pressure driving the emergence of resistant pathogens.

## Data Availability

Database will be open access available with the following https://doi.org/10.7303/syn61345336, or available from the corresponding author upon request. The data will be open access without limit time for those which have a synapse username. This criteria is asked by the Synapse page.

## Abbreviations

AMR: Antimicrobial resistance
ASPs: Antimicrobial stewardship programs
AMS: Antimicrobial stewardship
LATAM: Latin America
